# Investigation of Beraprost Sodium on Cardiac Function and Hemodynamics in Canine Models of Chronic Pulmonary Hypertension

**DOI:** 10.3389/fvets.2022.876178

**Published:** 2022-04-14

**Authors:** Ryohei Suzuki, Yunosuke Yuchi, Takahiro Saito, Takahiro Teshima, Hirotaka Matsumoto, Hidekazu Koyama

**Affiliations:** Laboratory of Veterinary Internal Medicine, School of Veterinary Medicine, Faculty of Veterinary Science, Nippon Veterinary and Life Science University, Musashino, Japan

**Keywords:** dog, myocardial function, pulmonary vascular resistance, pulmonary vasodilator, right ventricular strain, speckle tracking echocardiography, systemic vascular resistance

## Abstract

Pulmonary hypertension (PH) is a life-threatening disease in dogs characterized by increased pulmonary arterial pressure (PAP) and/or pulmonary vascular resistance. No study has evaluated the utility of Beraprost sodium (BPS) in dogs with PH. This study aimed to evaluate the effect of BPS on cardiac function and hemodynamics and examine the optimal dose of BPS in canine models of chronic embolic PH. In this prospective crossover study, three doses of BPS (5, 15, and 25 μg/kg, twice a day) were examined in eight canine models of chronic embolic PH. All model dogs underwent invasive PAP measurement, echocardiography, and non-invasive systemic blood pressure measurement before and after continuous administration of oral BPS for 1 week. No side effects of BPS were observed in any dog during the study. All doses of BPS significantly decreased systolic PAP and pulmonary vascular impedance. Additionally, systemic vascular impedance significantly decreased with 15 and 25 μg/kg of BPS. The right ventricular stroke volume and longitudinal strain significantly decreased with all doses of BPS. The left ventricular stroke volume and circumferential strain decreased with 15 μg/kg BPS. BPS was well-tolerated in this study. A dose-dependent vasodilating effect on pulmonary vessels was observed in canine models of chronic PH. Additionally, 15 μg/kg BPS showed a balanced vasodilating effect on systemic and pulmonary vessels. Furthermore, with a decrease in systemic and pulmonary vascular impedance, the left and right ventricular functions were significantly improved. Our results suggest that BPS may be useful in the treatment of canine PH.

## Introduction

Pulmonary hypertension (PH), a life-threatening disease in dogs, is characterized by increased pulmonary arterial pressure (PAP) and/or pulmonary vascular resistance (PVR) (1, 2). The disease induces right ventricular (RV) hypertrophy, dysfunction, and/or dilatation, and finally, right heart failure through increased RV afterload. In veterinary medicine, PH is commonly treated using phosphodiesterase-5 inhibitors, such as sildenafil, which selectively dilate pulmonary vessels by inactivating phosphodiesterase-5 and increasing cyclic guanosine monophosphate levels in the pulmonary vascular muscle. To date, various studies have reported the beneficial effects of sildenafil in dogs with PH (3–6). However, it might be clinically difficult to use sildenafil because of its high cost, difficulty in obtaining the drug, and the risk of excessive increase in pulmonary circulation due to its use, which might increase left heart load (7).

Beraprost sodium (BPS) is a chemically stable prostacyclin analog. BPS selectively activates adenylate cyclase, raises the cyclic adenosine monophosphate level in cells and blood platelets, and inhibits calcium ion influx and thromboxane-A_2_ production, resulting in vasodilating effects on pulmonary and systemic arterial smooth muscle cells, protective effects on vascular endothelial cells, inhibition of inflammatory cytokine production, and antiplatelet effects (8–11). In humans, BPS is commonly used to treat PH, and various studies have reported its efficacy in patients with PH (11–14). Additionally, BPS has been used to treat chronic kidney disease in cats (15). However, no study has reported the efficacy of BPS in dogs with PH. Furthermore, because of the species differences in susceptibility to prostacyclin, it is unclear whether the dose of BPS extrapolated to humans or felines is also appropriate in dogs with PH. Although there is only one study that has investigated the effect of intravenous administration of BPS in animal models of PH (11), no study has examined the optimal dose of oral BPS for the treatment of dogs with PH.

Therefore, the purpose of this study was to evaluate the effect of BPS on cardiac function and hemodynamics and to determine the optimal dose of BPS in canine models of chronic PH. We hypothesized that the vasodilating effect of BPS could increase dose-dependently and that BPS would also be useful in the treatment of canine PH.

## Materials and Methods

Our prospective, crossover study consisted of procedures that were performed in accordance with the Guide for Institutional Laboratory Animal Care and Use at Nippon Veterinary and Life Science University and was approved by the ethical committee for laboratory animal use of the Nippon Veterinary and Life Science University, Japan (approval number: 2019S-56).

### Animals

Eight laboratory-owned male beagles (body weight: 10.3 ± 0.5 kg, age: 2.2 ± 0.6 years) were used in this study. All dogs were regarded as clinically healthy following complete physical examination, blood tests, thoracic and abdominal radiography, transthoracic and abdominal ultrasonography, and oscillometry-derived blood pressure measurement.

### Creation of Chronic Embolic PH Model

All dogs were administered butorphanol tartrate (0.2 mg/kg, IV) (Meiji Seika Pharma Co. Ltd., Tokyo, Japan), midazolam hydrochloride (0.2 mg/kg, IV) (Maruishi Pharmaceutical. Co., Ltd., Osaka, Japan), heparin sodium (100 IU/kg, IV) (AY Pharmaceuticals Co. Ltd., Tokyo, Japan), and cefazolin sodium hydrate (20 mg/kg, IV) (LTL Pharma Co. Ltd., Tokyo, Japan). The dogs were anesthetized intravenously with propofol (Nichi-Iko Pharmaceutical Co., Ltd., Toyama, Japan) and maintained with 1.5–2.0% isoflurane (Mylan Seiyaku Ltd., Osaka, Japan) mixed with 100% oxygen. An ~5.0-cm surgical cutdown was performed over the right jugular furrow, and the right jugular vein was exteriorized. An 8-Fr multipurpose catheter tip (Atom Medical Corp., Tokyo, Japan) was placed in the main pulmonary artery under fluoroscopic guidance. After inserting the catheter, the catheter was fixed at the insertion site of the right jugular vein. Additionally, the catheter hub connecter was passed through the skin on the dorsal neck and was secured with a Chinese finger trap. Then, the neck was sutured, and all dogs completely recovered from anesthesia following conventional care (16).

Chronic PH was induced through continuous embolization of the peripheral pulmonary artery using microspheres measuring 150–300 μm in diameter (Sephadex G-25 Coarse; Cytiva, Tokyo, Japan) (17, 18). Approximately 0.1–0.4 g microspheres, which had been gas-sterilized in advance, were repeatedly injected into the main pulmonary artery through an 8-Fr multipurpose catheter twice a week. Embolic PH was defined as chronic if systolic PAP > 45 mmHg was maintained for >4 weeks without microsphere injection (17).

### Study Protocol

Three doses of BPS (5, 15, and 25 μg/kg, q12h) were tested using a crossover method in canine models with chronic PH, referencing previous toxicity studies using healthy dogs and studies using cats (15, 19–21). BPS was orally administered twice a day at each dose by dividing 55-μg tablets (Toray Industries, Inc., Tokyo, Japan) into appropriate doses for 1 week. The washout period for each dose was 1 week. Hemodynamic, echocardiography, and oscillometric blood pressure measurements were performed before and after continuous administration of BPS (pre and post). Each examination after continuous BPS administration was performed 2–3 h after BPS administration on day 7. The dogs were sedated using butorphanol tartrate (0.1 mg/kg, IV) and midazolam hydrochloride (0.1 mg/kg, IV) to perform each examination when necessary. After completing the study protocol, all dogs were then transferred to another protocol within our institution.

### Hemodynamic Assessment

Hemodynamic assessment was performed using circulatory function analysis software (SBP2000; Softron, Tokyo, Japan) (17). The dogs were restrained in right lateral recumbency, and PAP measurements (systolic, mean, and diastolic) and echocardiography were performed concurrently. The invasive PAP was measured following calibration with atmospheric pressure. The average PAP value was automatically calculated from nine consecutive cardiac cycles in the analysis software, and the obtained value was included in the statistical analysis.

In addition, systolic and mean systemic arterial pressures were obtained using an oscillometric method according to the ACVIM consensus statement (22). A cuff of appropriate size was wrapped around the ridge in all dogs to measure the systemic arterial pressure. Blood pressure measurements were performed repeatedly until three approximate values (<20% variability) were recorded. The average of the three approximate values was used for statistical analysis.

### Echocardiographic Assessment

Echocardiography was performed in all dogs concurrently with hemodynamic measurements before and after BPS administration. Two-dimensional and Doppler examinations were performed by a single investigator (RS) using a Vivid 7 or Vivid E95 echocardiographic system (GE Healthcare, Tokyo, Japan) and a 3.5–6.9-MHz transducer. A lead II electrocardiogram was recorded simultaneously, and the images were displayed. The dogs were manually restrained in right and left lateral recumbency, and data were obtained from at least five consecutive cardiac cycles in sinus rhythm. Images were analyzed using an offline workstation (EchoPAC PC, Version 204; GE Healthcare, Tokyo, Japan) by a single observer (YY). The mean of three consecutive cardiac cycles were used for statistical analyses.

In this study, end-diastolic and end-systolic left ventricular (LV) internal dimensions (LVIDd and LVIDs, respectively), end-diastolic and end-systolic LV volumes obtained using the biplane modified Simpson's method (LVEDV and LVESV, respectively), and left atrial-to-aortic diameter ratio (LA/Ao) were measured as indicators of left heart morphology using the B-mode method, as described previously (23–26). LVIDd and LVIDs were normalized by body weight (LVIDDN and LVIDSN, respectively), and LVEDV and LVESV were normalized by body surface area (LVEDVI and LVESVI, respectively). The body surface area was calculated using the following formula (27).


$$\text{Body surface area }\left({\text{m}}^{\text{2}}\right)\text{ = 0}.\text{101​}\times \text{ body }{\text{weight}}^{\text{2/3}}$$

Additionally, LV fractional shortening and ejection fraction (EF) were calculated using LV internal dimension and volume, respectively (26).

The end-diastolic and end-systolic RV areas normalized by body weight (RVEDA index and RVESA index, respectively) were measured as RV size indicators using the left apical four-chamber view optimized for the right heart (RV focus view), as described previously (28–30). Additionally, the ratio of pulmonary artery-to-aortic diameter (PA/Ao) was obtained from the right parasternal short-axis view at the level of the pulmonary artery, as described previously (31).

For RV functional assessment, tricuspid annular plane systolic excursion (TAPSE), RV fractional area change (RV FAC), and tissue Doppler imaging-derived peak systolic myocardial velocity of the lateral tricuspid annulus (RV s′) were measured (28–30). All RV functional variables were obtained using the RV focus view. TAPSE was measured using the B-mode method as previously described (32–34). TAPSE and RV FAC were also normalized by body weight, as described previously (TAPSEn and RV FACn, respectively) (17, 28, 34). The RV s′ was obtained from the tissue Doppler imaging-derived lateral tricuspid annular motion wave.

Systemic arterial impedance (SVI) and pulmonary arterial impedance (PVI) were calculated as the indicator of systemic vascular resistance and PVR, respectively. SVI was calculated by dividing systolic systemic arterial pressure obtained from the oscillometric method by LV stroke volume normalized by the body surface area (LV SV). PVI was calculated by dividing invasive systolic PAP by RV stroke volume normalized by body surface area (RV SV) (35). LV SV and RV SV were measured using the cross-sectional area method as described previously (36). Additionally, cardiac output of left and right ventricle normalized by body surface area (LV CO and RV CO, respectively) were calculated using SV and heart rate calculated by mean R-R intervals obtained from the same cardiac cycle used for each SV measurement.

### Two-Dimensional Speckle Tracking Echocardiography

In this study, two-dimensional speckle tracking echocardiography (2D-STE) was performed as a precise myocardial function indicator of the left and right ventricles. All 2D-STE analyses were performed by a single investigator (YY) using the same offline workstation as that used for standard echocardiography. The strain was obtained from the right parasternal short-axis view at the level of the papillary muscle for the LV circumferential strain (LV-SC), left apical four-chamber view for the LV longitudinal strain (LV-SL), and RV focus view for the RV longitudinal strain (RV-SL). The region of interest for 2D-STE was defined by manually tracing the endocardial borders of the left and right ventricles. RV-SL was obtained using two methods and the left ventricular four-chamber algorithm (17, 32, 37, 38). Only RV free wall analysis (3seg) was performed by tracing from the level of the lateral tricuspid annulus to the RV apex, whereas RV global analysis (6seg) was also performed by tracing from the lateral tricuspid annulus to the septal tricuspid annulus (including the interventricular septum) via the RV apex. Manual adjustments were made when necessary to include and track the entire myocardial thickness over the cardiac cycle. All strains were defined as the absolute value of the negative peak obtained from each strain wave (32, 38, 39). The mean of three consecutive cardiac cycles were used for statistical analyses.

### Statistical Analysis

All statistical analyses were performed using commercially available EZR software, version 1.41 (Saitama Medical Center, Jichi Medical University, Saitama, Japan) (40). All continuous data are reported as mean ± standard deviation. Additionally, the rate of change before and after BPS administration were calculated for SVI and PVI.

The Shapiro–Wilk test was performed to evaluate the normality of the data. Continuous variables were compared between pre- and post-examination using the paired *t*-test for normally distributed data or Wilcoxon signed rank-sum test for non-normally distributed data. Additionally, to evaluate whether 1 week washout period was sufficient or not, PAP at baseline of each BPS dose were compared using one-way analysis of variance with subsequent pairwise comparisons using Tukey's multiple comparison test for normally distributed data or the Kruskal–Wallis test with subsequent pairwise comparisons using the Steel–Dwass test for non-normally distributed data. Statistical significance was set at *p* < 0.050.

## Results

All dogs were repeatedly injected with the microsphere for 50.9 ± 13.1 weeks to meet the definition of chronic PH. The median total dose of microspheres was 1.24 g/kg (range: 0.93–1.37) (17). All dogs completed the study protocol, and no side effects of BPS, such as hypotension or abnormal hemostasis, were observed in any dogs during the study protocol. Only one dog needs sedation throughout the study protocol. At study inclusion, the mean ± standard deviation of systolic, mean, and diastolic PAP of all dogs were 52.5 ± 7.4, 31.4 ± 3.3, 17.5 ± 3.4, respectively. Additionally, no abnormal values were measured in systolic and mean systemic blood pressure in any dogs at study inclusion (mean ± standard deviation: 129.1 ± 7.8 and 92.7 ± 10.1 mmHg, respectively). The mean RVEDA index, RVESA index, and PA/Ao were over and the mean RV FACn and 2D-STE derived RV-SL were under the reference intervals reported previously (Table 1) (28, 34, 41). There were no significant abnormalities in echocardiographic indices of LV morphology or function at study inclusion. There were no significant differences in systolic, mean, and diastolic PAP at baseline of each BPS dose (*p* = 0.184, 0.254, and 0.554, respectively).

**Table 1:** Results of echocardiographic variables of eight canine models of chronic pulmonary hypertension at study inclusion.

**Variables for left heart**		**Variables for right heart**	
LA/Ao	1.0 ± 0.1	PA/Ao	0.93 ± 0.1
LVIDDN (cm/kg^0.297^)	1.4 ± 0.2	RVEDA index (cm^2^/kg^0.624^)	1.3 ± 0.2
LVIDSN (cm/kg^0.315^)	0.9 ± 0.2	RVESA index (cm^2^/kg^0.628^)	0.9 ± 0.3
FS (%)	41.9 ± 6.6	RVIDd index (mm/kg^0.327^)	8.5 ± 1.5
LVEDVI (mL/m^2^)	33.3 ± 6.7	TAPSEn (mm/kg^0.284^)	5.6 ± 0.9
LVESVI (mL/m^2^)	16.9 ± 4.2	RV FACn (%/kg^−0.097^)	39.4 ± 11.3
EF (%)	49.4 ± 4.4	RV s' (cm/s)	10.7 ± 2.6
LV SV (mL/m^2^)	37.7 ± 5.9	RV SV (mL/m^2^)	46.5 ± 11.1
LV CO (L/min/m^2^)	3.5 ± 0.8	RV CO (L/min/m^2^)	3.9 ± 1.1
LV-SL (%)	14.3 ± 1.9	RV-SL_3seg_ (%)	20.5 ± 2.8
LV-SrL (%/s)	1.6 ± 0.3	RV-SL_6seg_ (%)	17.7 ± 3.0
LV-SC (%)	19.8 ± 3.4	RV-SrL_3seg_ (%/s)	2.5 ± 0.6
LV-SrC (%/s)	2.0 ± 0.4	RV-SrL_6seg_ (%/s)	2.1 ± 0.6

*3seg, only right ventricular free wall analysis; 6seg, right ventricular global analysis; CO, cardiac output normalized by body surface area; EF, ejection fraction; LA/Ao, left atrium to aortic diameter ratio; LV, left ventricular; LVEDVI, end-diastolic LV volume normalized by body surface area; LVESVI, end-systolic LV volume normalized by body surface area; LVIDDN, end-diastolic LV internal dimension normalized by body weight; LVIDSN, end-systolic LV internal dimension normalized by body weight; LV-SC; LV circumferential strain; LV-SL, LV longitudinal strain; LV-SrC, LV circumferential strain rate; LV-SrL, LV longitudinal strain rate; PA/Ao, pulmonary artery to aortic diameter ratio; RV, right ventricular; RV FACn, RV fractional area change normalized by body weight; RV s', tissue Doppler imaging-derived peak systolic myocardial velocity of lateral tricuspid annulus; RVEDA index, end-diastolic RV area normalized by body weight; RVESA index, end-systolic RV area normalized by body weight; RVIDd index, end-diastolic RV internal dimension normalized by body weight; RV-SL, RV longitudinal strain; RV-SrL, RV longitudinal strain rate; RVWTd, end-diastolic RV wall thickness; SV, stroke volume normalized by body surface area; TAPSEn, tricuspid annular plane systolic excursion normalized by body weight*.

*Continuous variables were displayed as mean ± standard deviation*.

### Hemodynamic Measurements

**Figure 1:**
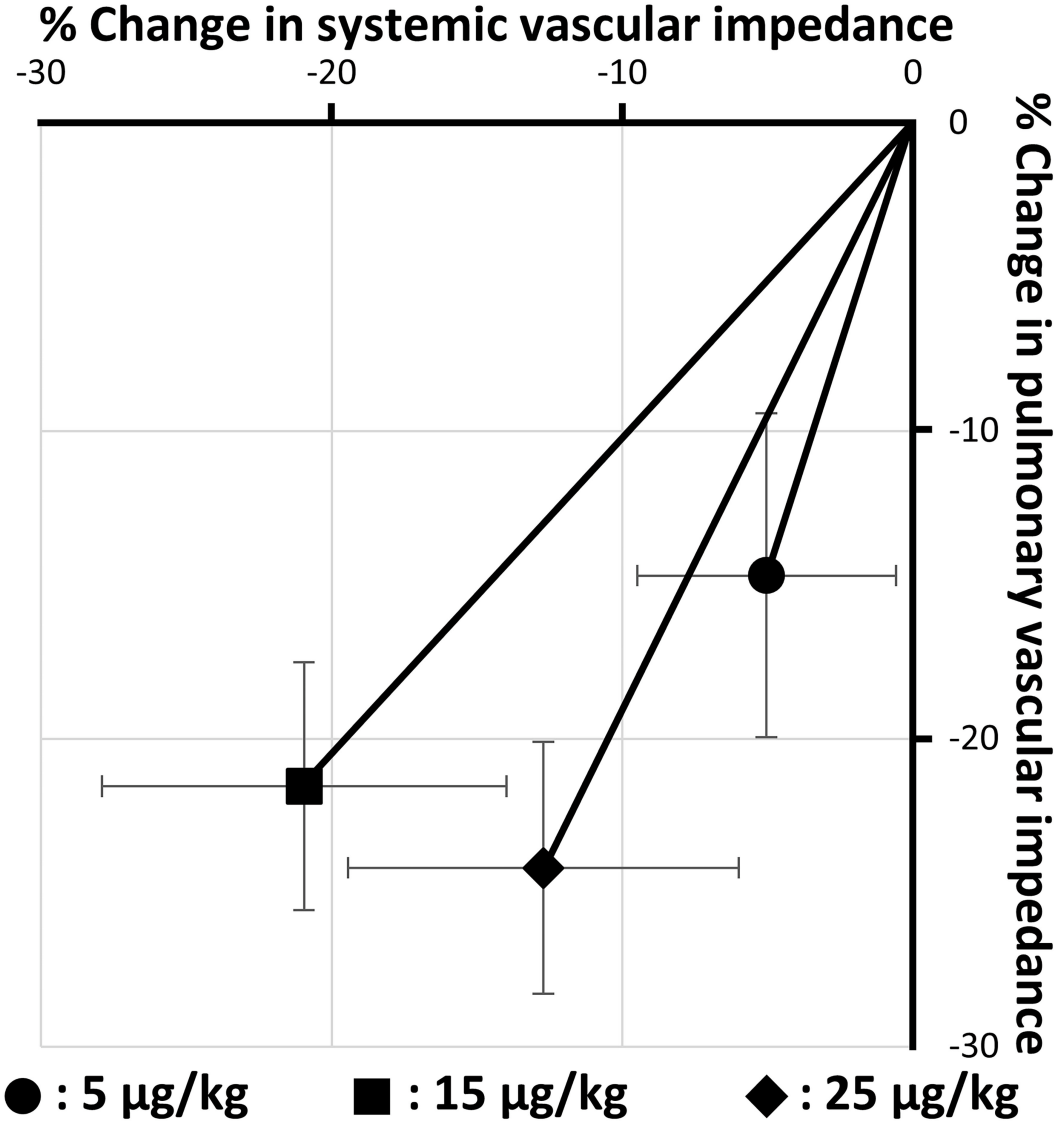
Changes in pulmonary and systemic vascular impedance by three doses of beraprost sodium administration (5, 15, and 25 μg/kg). Values are the means ± standard deviations.

Table 2 shows the changes in hemodynamic variables before and after BPS administration. In this study, all doses of BPS decreased systolic PAP (5 μg/kg, *p* = 0.034; 15 μg/kg, *p* = 0.035; and 25 μg/kg, *p* = 0.047). The mean PAP decreased with the administration of 15 and 25 μg/kg BPS (*p* = 0.016 and *p* = 0.018, respectively). Diastolic PAP showed no significant change following BPS administration. Additionally, PVI significantly decreased with all doses of BPS (5 μg/kg, *p* = 0.020; 15 μg/kg, *p* = 0.019; 25 μg/kg, *p* = 0.003). No significant changes were observed in systemic blood pressure obtained using the oscillometric method. However, SVI significantly decreased with the administration of 15 and 25 μg/kg BPS (*p* = 0.005 and *p* = 0.041, respectively). Based on the percentage change in PVI and SVI represented in Figure 1, BPS decreased PVI in a dose-dependent manner. Furthermore, only 15 μg/kg of BPS decreased PVI and SVI equally. In contrast, 5 and 25 μg/kg BPS had a vasodilating effect that was selective for pulmonary vessels.

**Table 2:** Changes in hemodynamic parameters before and after beraprost sodium administration.

**Variables**	**BPS 5 μg/kg**		**BPS 15 μg/kg**		**BPS 25 μg/kg**	
	**Pre**	**Post**	**Pre**	**Post**	**Pre**	**Post**
**Pulmonary arterial pressure**						
Systole (mmHg)	53.2 ± 9.5	50.3 ± 8.9^*^	52.3 ± 7.0	47.1 ± 4.1^*^	50.8 ± 6.7	46.4 ± 6.7^*^
Mean (mmHg)	31.3 ± 3.8	31.1 ± 3.4	31.3 ± 3.2	28.2 ± 3.4^*^	30.9 ± 2.7	28.8 ± 3.7^*^
Diastole (mmHg)	16.3 ± 4.3	18.8 ± 5.2	16.9 ± 4.2	15.8 ± 3.3	18.5 ± 3.0	17.4 ± 5.1
PVI (mmHg/mL)	1.2 ± 0.3	1.0 ± 0.2^*^	1.2 ± 0.3	0.9 ± 0.1^*^	1.1 ± 0.2	0.8 ± 0.1^*^
**Systemic arterial pressure**						
Systole (mmHg)	130 ± 9	128 ± 10	131 ± 11	126 ± 8	127 ± 9	121 ± 11
Mean (mmHg)	97 ± 10	98 ± 9	97 ± 9	90 ± 10	92 ± 9	85 ± 10
SVI (mmHg/mL)	3.4 ± 0.7	3.2 ± 0.6	3.5 ± 0.7	2.8 ± 0.5^*^	3.0 ± 0.5	2.6 ± 0.6^*^
Heart rate (bpm)	90 ± 11	94 ± 18	89 ± 14	90 ± 11	85 ± 16	93 ± 16

*BPS, beraprost sodium; PVI, pulmonary vascular impedance; SVI, systemic vascular impedance*.

*Continuous variables were displayed as mean ± standard deviation*.

* *The value is significantly different from pre-examination of the corresponding dose of BPS (p < 0.050)*.

### Echocardiographic Measurements

**Figure 2:**
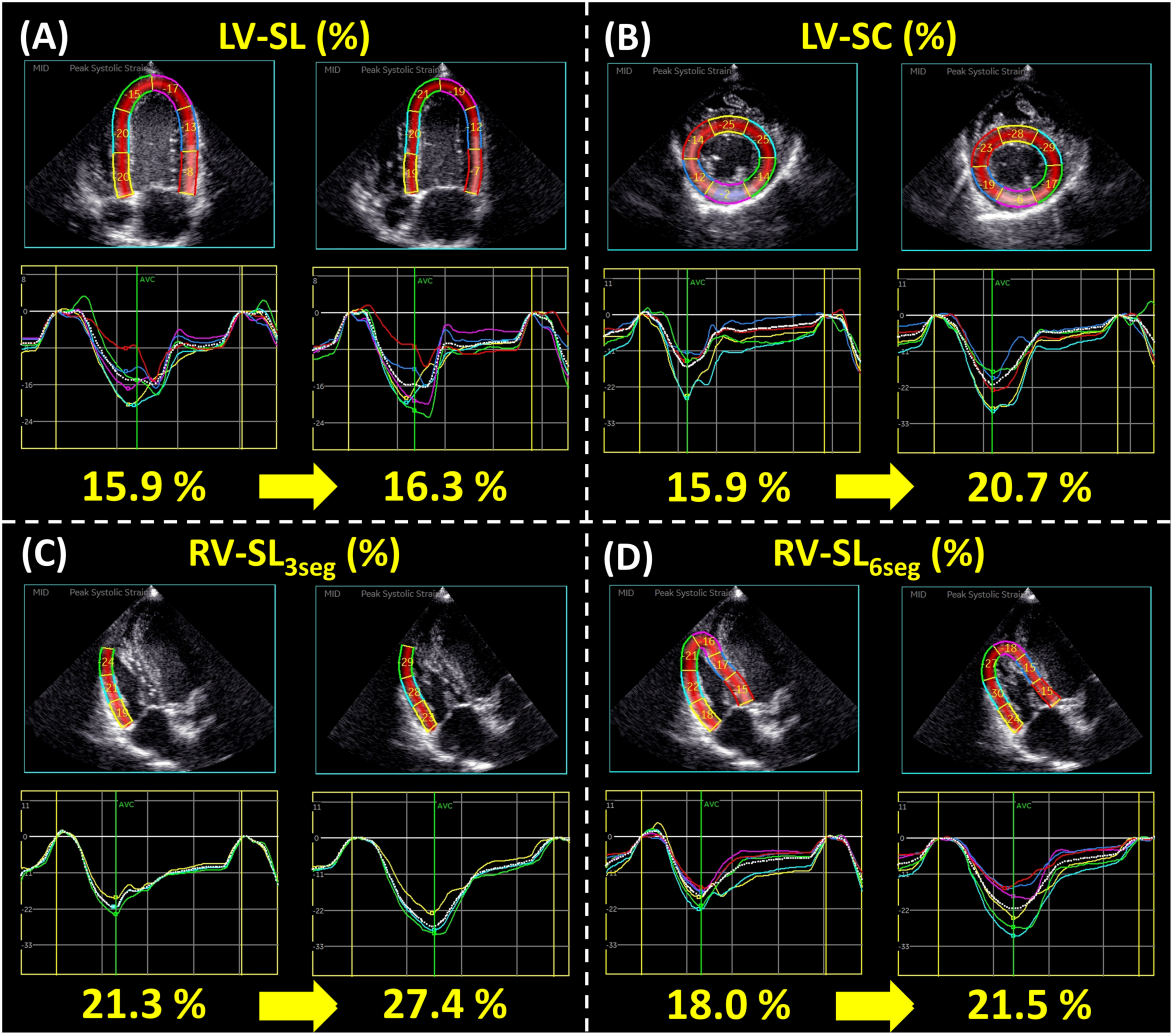
Representative data of two-dimensional speckle tracking echocardiography-derived myocardial strain before and after 15 μg/kg beraprost sodium (BPS) administration: left ventricular longitudinal strain [LV-SL **(A)**], left ventricular circumferential strain [LV-SC **(B)**], right ventricular longitudinal strain obtained from only right ventricular free wall analysis and right ventricular global analysis [RV-SL_3seg_ **(C)** and RV-SL_6seg_ **(D)**, respectively]. For each variable, the left figure represents the myocardial strain before BPS administration, and the right represents that after BPS administration.

Changes in echocardiographic variables for LV morphology and function are summarized in Table 3. Regarding LV morphology, there were no significant differences in LA/Ao, LV internal dimension, LV volume, or 2D-STE-derived LV-SL and LV-SrL before and after BPS administration (Figure 2A). EF and LV SV significantly increased with administration of 15 and 25 μg/kg BPS (EF: both *p* < 0.001, LV SV: *p* = 0.004, and *p* = 0.012, respectively). Additionally, LV CO, 2D-STE-derived LV-SC, and LV-SrC showed significant increases with 15 μg/kg BPS administration (*p* = 0.007, *p* = 0.007, and *p* = 0.006, respectively) (Figure 2B).

**Table 3:** Changes in echocardiographic parameters for left heart morphology and function before and after beraprost sodium administration.

**Variables**	**BPS 5 μg/kg**		**BPS 15 μg/kg**		**BPS 25 μg/kg**	
	**Pre**	**Post**	**Pre**	**Post**	**Pre**	**Post**
LA/Ao	1.0 ± 0.1	1.0 ± 0.1	1.0 ± 0.1	1.1 ± 0.0	1.0 ± 0.1	1.1 ± 0.1
LVIDDN (cm/kg^0.297^)	1.6 ± 0.1	1.6 ± 0.1	1.6 ± 0.1	1.6 ± 0.2	1.6 ± 0.1	1.5 ± 0.1
LVIDSN (cm/kg^0.315^)	1.0 ± 0.1	1.0 ± 0.1	1.0 ± 0.1	0.9 ± 0.2	0.9 ± 0.2	0.9 ± 0.2
FS (%)	38.3 ± 4.6	37.5 ± 4.6	35.6 ± 6.3	37.6 ± 11.4	40.3 ± 7.5	40.9 ± 7.4
LVEDVI (mL/m^2^)	65.7 ± 7.4	64.1 ± 7.3	71.1 ± 11.8	71.7 ± 9.6	70.2 ± 12.4	70.5 ± 9.7
LVESVI (mL/m^2^)	35.0 ± 6.1	33.0 ± 6.3	37.2 ± 5.7	34.2 ± 5.3	36.3 ± 7.5	33.0 ± 6.6^*^
EF (%)	46.7 ± 6.8	48.8 ± 6.1	47.2 ± 2.4	52.6 ± 2.9^*^	48.1 ± 3.8	53.3 ± 4.2^*^
LV SV (mL/m^2^)	39.4 ± 7.6	40.9 ± 7.3	37.7 ± 4.9	46.4 ± 5.6^*^	43.1 ± 6.8	48.3 ± 11.1^*^
LV CO (L/min/m^2^)	3.8 ± 0.7	3.9 ± 0.7	3.3 ± 0.5	4.5 ± 0.7^*^	4.1 ± 0.6	4.7 ± 1.1
LV-SL (%)	13.8 ± 0.9	13.7 ± 1.2	13.5 ± 1.7	14.7 ± 2.0	14.4 ± 2.3	14.5 ± 1.8
LV-SrL (%/s)	1.6 ± 0.2	1.5 ± 0.2	1.4 ± 0.2	1.5 ± 0.2	1.6 ± 0.4	1.6 ± 0.2
LV-SC (%)	19.1 ± 1.7	19.0 ± 1.5	16.6 ± 2.4	18.8 ± 1.8^*^	19.2 ± 3.1	20.4 ± 1.3
LV-SrC (%/s)	1.9 ± 0.2	1.9 ± 0.2	1.7 ± 0.2	1.9 ± 0.2^*^	2.0 ± 0.4	2.0 ± 0.1

*BPS, beraprost sodium; COI, cardiac output normalized by body surface area; EF, ejection fraction; LA/Ao, left atrium to aortic diameter ratio; LV, left ventricular; LVEDVI, end-diastolic left ventricular volume normalized by body surface area; LVESVI, end-systolic left ventricular volume normalized by body surface area; LVIDDN, end-diastolic left ventricular internal dimension normalized by body weight; LVIDSN, end-systolic left ventricular internal dimension normalized by body weight; LV-SC; left ventricular circumferential strain; LV-SL, LV longitudinal strain; LV-SrC, LV circumferential strain rate; LV-SrL, LV longitudinal strain rate; SVI, stroke volume normalized by body surface area*.

*Continuous variables were displayed as mean ± standard deviation*.

* *The value is significantly different from pre-examination of the corresponding dose of BPS (p < 0.050)*.

Table 4 shows the changes in echocardiographic variables of RV morphology and function before and after BPS administration. Although, there were no significant changes in PA/Ao, RV area, RVIDd, or RV s′ in this study, TAPSEn, RV SV, 2D-STE-derived RV-SL_3seg_, and RV-SL_6seg_ were significantly increased by all doses of BPS (Figures 2C,D). Additionally, RV FACn and RV CO were significantly increased with administration of 15 and 25 μg/kg BPS (RV FACn: *p* = 0.003 and *p* = 0.007, respectively, RV CO: *p* = 0.012 and *p* = 0.025, respectively).

**Table 4:** Changes in echocardiographic parameters for right heart morphology and function before and after beraprost sodium administration.

**Variables**	**BPS 5 μg/kg**		**BPS 15 μg/kg**		**BPS 25 μg/kg**	
	**Pre**	**Post**	**Pre**	**Post**	**Pre**	**Post**
PA/Ao	0.9 ± 0.1	0.9 ± 0.1	1.0 ± 0.0	1.0 ± 0.1	0.9 ± 0.1	0.9 ± 0.1
RVEDA index (cm^2^/kg^0.624^)	1.3 ± 0.2	1.4 ± 0.2	1.4 ± 0.3	1.4 ± 0.3	1.3 ± 0.3	1.4 ± 0.2
RVESA index (cm^2^/kg^0.628^)	0.9 ± 0.2	0.9 ± 0.2	1.0 ± 0.2	0.9 ± 0.2	0.9 ± 0.2	0.9 ± 0.2
RVIDd index (mm/kg^0.327^)	18.6 ± 3.0	19.5 ± 3.4	19.0 ± 4.0	18.4 ± 3.0	18.2 ± 2.6	18.7 ± 3.9
TAPSEn (mm/kg^0.284^)	5.6 ± 0.8	6.0 ± 0.9^*^	5.3 ± 0.8	6.1 ± 0.7^*^	5.7 ± 0.9	6.2 ± 1.1^*^
RV FACn (%/kg^−0.097^)	37.9 ± 8.8	41.1 ± 5.9	38.8 ± 8.4	45.9 ± 7.1^*^	40.3 ± 9.0	45.4 ± 7.3^*^
RV s' (cm/s)	9.6 ± 2.5	10.9 ± 2.2	10.1 ± 2.5	10.9 ± 1.9	11.6 ± 2.3	10.4 ± 2
RV SV (mL/m^2^)	48.3 ± 12.7	53.0 ± 10.5^*^	45 ± 12.7	54.5 ± 13.8^*^	48.1 ± 8.9	56.8 ± 12.8^*^
RV CO (L/min/m^2^)	4.3 ± 1.3	5.0 ± 1.5	3.9 ± 0.8	4.9 ± 1.6^*^	4.1 ± 1.0	5.1 ± 2.1^*^
RV-SL_3seg_ (%)	19.1 ± 3.3	22.4 ± 3.7^*^	21.1 ± 4.2	23.1 ± 4.0^*^	18.6 ± 3.8	20.7 ± 4.4^*^
RV-SL_6seg_ (%)	15.4 ± 3.1	18.2 ± 2.4^*^	17.0 ± 3.5	18.8 ± 2.9^*^	15.8 ± 3.8	17.1 ± 3.0^*^
RV-SrL_3seg_ (%/s)	2.4 ± 0.7	2.4 ± 0.6	2.2 ± 0.6	2.5 ± 0.7	2.3 ± 0.5	2.3 ± 0.5
RV-SrL_6seg_ (%/s)	1.9 ± 0.5	1.9 ± 0.3	1.8 ± 0.4	2.1 ± 0.4^*^	1.9 ± 0.5	1.9 ± 0.4

*3seg, only right ventricular free wall analysis; 6seg, right ventricular global analysis; BPS, beraprost sodium; CO, cardiac output normalized by body surface area; PA/Ao, pulmonary artery to aortic diameter ratio; RV, right ventricular; RV FACn, RV fractional area change normalized by body weight; RV s', tissue Doppler imaging-derived peak systolic myocardial velocity of lateral tricuspid annulus; RVEDA index, end-diastolic RV area normalized by body weight; RVESA index, end-systolic RV area normalized by body weight; RVIDd index, end-diastolic RV internal dimension normalized by body weight; RV-SL, RV longitudinal strain; RV-SrL, RV longitudinal strain rate; RVWTd, end-diastolic RV wall thickness; SV, stroke volume normalized by body surface area; TAPSEn, tricuspid annular plane systolic excursion normalized by body weight*.

*Continuous variables were displayed as mean ± standard deviation*.

* *The value is significantly different from pre-examination of the corresponding dose of BPS (p < 0.050)*.

## Discussion

This is the first study to evaluate the optimal dose of oral BPS in canine models of chronic PH. In this study, all doses of BPS significantly decreased systolic PAP and PVI, and 15 and 25 μg/kg of BPS decreased SVI. Our results suggest that BPS had a dose-dependent effect on pulmonic and systemic vasodilatation, and 15 μg/kg BPS showed the most balanced vasodilatory effect. Additionally, with the decrease in PVI and SVI, PAP, LV, and RV function based on EF, 2D-STE-derived LV-SC and RV-SL were significantly improved. These results suggest that high-dose oral BPS may be an additional treatment option for dogs with PH.

In this study, the pulmonary vasodilating effect increased with an increase in BPS dose. These results agree with those of a previous study that reported the utility of intravenous BPS injection in dogs with PH induced by a thromboxane-A_2_ agonist (11). Our results suggest that oral BPS may be useful for the treatment of canine PH and that BPS might decrease PAP in a dose-dependent manner. Additionally, although not evaluated in this study, the antiplatelet effect of BPS might also be effective in diseases that might increase PAP due to pulmonary arterial thromboembolism, such as immune-mediated hemolytic anemia, spontaneous hyperadrenocorticism, protein-losing nephropathy, and disseminated intravascular coagulation syndrome (1).

This study compared the effects of three doses of BPS (5, 15, and 25 μg/kg) on right heart function and hemodynamics. Although BPS decreased PAP in a dose-dependent manner, the selection of the maximal BPS dose was set at 25 μg/kg according to the previous toxicity studies that have reported that the no-observed-adverse-effect level was 25 μg/kg (19, 20). In humans, the recommended dose of BPS for the treatment of PH is 60–180 μg/day (i.e., 1–3 μg/kg/day for a 60 kg person) (11–14). Although systolic PAP and PVI in our study were significantly decreased with all doses of BPS administration, the mean PAP results suggest that a higher dose of BPS may be needed for the treatment of PH in dogs than that in humans, possibly due to species differences in the effects of BPS. Additionally, our results suggest that up to 25 μg/kg BPS could be safely used in dogs with PH without any side effects.

A significant decrease in SVI without an excessive decrease in systemic blood pressure was observed with administration of 15 and 25 μg/kg BPS. Additionally, 15 μg/kg BPS showed the most balanced decrease in PVI and SVI (Figure 1). A previous study has reported that the vasodilating effect, which predominantly affects pulmonary vessels over systemic vessels, might increase left heart load (7). In veterinary medicine, the most common cause of PH is mitral valve insufficiency (2, 42, 43). Sildenafil and 5 μg/kg of BPS, which is highly selective for pulmonary vessels, might worsen left heart disease, especially in dogs with PH, due to increased left atrial pressure. Additionally, 25 μg/kg BPS was also observed to have an imbalanced vasodilating effect. This was possibly because the systemic vasodilating effect might have peaked at a lower dose than the pulmonary vasodilating effect. Therefore, 15 μg/kg BPS may be the optimal dose for the treatment of canine PH. However, 25 μg/kg BPS might be needed in cases with decreased left heart size due to excessively decreased pulmonary circulation. Further studies involving dogs with underfilled left heart volume due to severe PH are expected to validate the clinical efficacy of 25 μg/kg BPS in dogs with PH.

No studies have investigated the effect of BPS on RV function in dogs with PH. In this study, significant improvements were observed in various echocardiographic indices of RV function, including the TAPSEn, RV FACn, RV SV, RV CO, and 2D-STE-derived RV-SL. Especially, 2D-STE variables enable precise myocardial function assessment with angle independence and minimal influence of loading conditions (44). Our results suggest that a high dose of BPS could improve RV function and pulmonary circulation, possibly through decreases in PVR and PAP (i.e., RV afterload). Additionally, LV function based on EF, 2D-STE-derived LV-SC, LV SV, and LV CO also improved with 15 μg/kg of BPS administration. Our EF results were consistent with those of a previous study on patients with PH secondary to left heart disease (14). Although almost all dogs showed no significant changes in LV morphology, the improvement in pulmonary circulation and decrease in LV afterload might have led to the results. Therefore, administration of a high dose of BPS might improve LV function and systemic circulation, as well as RV function and pulmonary circulation. Further studies including dogs with PH secondary to left heart disease should be conducted to ensure the safety and efficacy of BPS in dogs with PH.

This study had several limitations. First, this study used canine models of chronic embolic PH. Drug responsiveness to BPS might differ in clinical cases of PH and PH with different causes, such as left heart disease and respiratory disease. Second, the dose frequency of BPS was limited to twice-daily in this study. BPS administration three times per day is common in human PH (11–14). Therefore, more frequent administration of BPS might be effective in dogs with PH. Finally, we did not perform a priori power calculations. The relatively small sample size may have had insufficient power to detect differences.

In conclusion, oral administration of BPS significantly decreased PVI, systolic PAP, and SVI in a dose-dependent manner. Along with the decrease in PVI and SVI, significant improvements were observed in various echocardiographic indices of LV and RV function, including EF and 2D-STE-derived LV-SC and RV-SL. The 15 μg/kg of BPS showed the most balanced decreases in SVI and PVI, whereas 5 and 25 μg/kg of BPS showed a vasodilating effect selective for pulmonary vessels. Our results suggest that oral BPS might be an additional treatment option for dogs with PH, and 15 μg/kg might be the optimal dose for canine PH treatment. Further studies that include dogs with PH due to various causes are needed to validate the clinical utility of BPS in dogs with PH.

## Data Availability Statement

The raw data supporting the conclusions of this article will be made available by the authors, without undue reservation.

## Ethics Statement

The animal study was reviewed and approved by Ethical Committee for Laboratory Animal Use of Nippon Veterinary and Life Science University, Japan.

## Author Contributions

RS conceived of the academic direction, performed conceptualization and design of the study, data analysis, interpretation, and critical revision of the article, and approved the article. YY performed conceptualization and design of the study, data analysis, interpretation, article drafting, and critical revision of the article. TS, TT, HM, and HK interpreted the data, critically revised the manuscript, and approved the manuscript. All authors contributed to the article and approved the submitted version.

## Funding

This work was partially supported by the Japan Society for the Promotion of Science (JSPS) KAKENHI, Grant No. 20K15667.

## Conflict of Interest

HK received a grant from Toray Industries, Inc. The remaining authors declare that the research was conducted in the absence of any commercial or financial relationships that could be construed as a potential conflict of interest.

## Publisher's Note

All claims expressed in this article are solely those of the authors and do not necessarily represent those of their affiliated organizations, or those of the publisher, the editors and the reviewers. Any product that may be evaluated in this article, or claim that may be made by its manufacturer, is not guaranteed or endorsed by the publisher.

## Abbreviations

2D-STE: two-dimensional speckle tracking echocardiography
3seg: only right ventricular free wall analysis
6seg: right ventricular global analysis
BPS: beraprost sodium
CO: cardiac output normalized by body surface area
EF: ejection fraction
LA/Ao: left atrium-to-aortic diameter ratio
LV: left ventricle/left ventricular
LVEDVI: end-diastolic LV volume normalized by body surface area
LVESVI: end-systolic LV volume normalized by body surface area
LVIDDN: end-diastolic LV internal dimension normalized by body weight
LVIDSN: end-systolic LV internal dimension normalized by body weight
LV-SC: LV circumferential strain
LV-SL: LV longitudinal strain
LV-SrC: LV circumferential strain rate
LV-SrL: LV longitudinal strain rate
PA/Ao: pulmonary artery-to-aortic diameter ratio
PAP: pulmonary arterial pressure
PH: pulmonary hypertension
PVI: pulmonary vascular impedance
PVR: pulmonary vascular resistance
RV: right ventricle/right ventricular
RV FACn: RV fractional area change normalized by body weight
RV s′: tissue Doppler imaging-derived peak systolic myocardial velocity of lateral tricuspid annulus
RVEDA index: end-diastolic RV area normalized by body weight
RVESA index: end-systolic RV area normalized by body weight
RVIDd index: end-diastolic RV internal dimension normalized by body weight
RV-SL: RV longitudinal strain
RV-SrL: RV longitudinal strain rate
SV: stroke volume normalized by body surface area
SVI: systemic vascular impedance
TAPSEn: tricuspid annular plane systolic excursion normalized by body weight.

## References

[1] Reinero C Visser LC Kellihan HB Masseau I Rozanski E Clercx C. ACVIM consensus statement guidelines for the diagnosis, classification, treatment, and monitoring of pulmonary hypertension in dogs. J Vet Intern Med. (2020) 34:549–73. 10.1111/jvim.15725 32065428 PMC7097566

[2] Johnson LR Boon J Orton EC. Clinical characteristics of 53 dogs with Doppler-derived evidence of pulmonary hypertension: 1992-1996. J Vet Intern Med. (1999) 13:440–7. 10.1111/j.1939-1676.1999.tb01461.x 10499728

[3] Akabane R Sakatani A Ogawa M Nagakawa M Miyakawa H Miyagawa Y. The effect of sildenafil on pulmonary haemodynamics in a canine model of chronic embolic pulmonary hypertension. Res Vet Sci. (2020) 133:106–10. 10.1016/j.rvsc.2020.08.019 32961474

[4] Johnson LR Stern JA. Clinical features and outcome in 25 dogs with respiratory-associated pulmonary hypertension treated with sildenafil. J Vet Intern Med. (2020) 34:65–73. 10.1111/jvim.15679 31816127 PMC6979098

[5] Kellihan HB Waller KR Pinkos A Steinberg H Bates ML. Acute resolution of pulmonary alveolar infiltrates in 10 dogs with pulmonary hypertension treated with sildenafil citrate: 2005-2014. J Vet Cardiol. (2015) 17:182–91. 10.1016/j.jvc.2015.04.002 26293206

[6] Bach JF Rozanski EA MacGregor J Betkowski JM Rush JE. Retrospective evaluation of sildenafil citrate as a therapy for pulmonary hypertension in dogs. J Vet Intern Med. (2006) 20:1132–5. 10.1111/j.1939-1676.2006.tb00711.x 17063705

[7] Hoendermis ES Liu LCY Hummel YM Van Der Meer P De Boer RA Berger RMF. Effects of sildenafil on invasive haemodynamics and exercise capacity in heart failure patients with preserved ejection fraction and pulmonary hypertension: a randomized controlled trial. Eur Heart J. (2015) 36:2565–73. 10.1093/eurheartj/ehv336 26188003

[8] Koh E Morimoto S Jiang B Inoue T Nabata T Kitano S. Effects of beraprost sodium, a stable analogue of prostacyclin, on hyperplasia, hypertrophy and glycosaminoglycan synthesis of rat aortic smooth muscle cells. Artery. (1993) 20:242–52. 8141645

[9] Akiba T Miyazaki M Toda N. Vasodilator actions of TRK-100, a new prostaglandin I2 analogue. Br J Pharmacol. (1986) 89:703–11. 10.1111/j.1476-5381.1986.tb11174.x 3101928 PMC1917240

[10] Nishio S Matsuura H Kanai N Fukatsu Y Hirano T Nishikawa N. The *in vitro* and *ex vivo* antiplatelet effect of TRK-100, a stable prostacyclin analog, in several species. Jpn J Pharmacol. (1988) 47:1–10. 10.1016/S0021-5198(19)43244-7 2842529

[11] Tamura M Kurumatani H Matsushita T. Comparative effects of beraprost, a stable analogue of prostacyclin, with PGE1, nitroglycerin and nifedipine on canine model of vasoconstrictive pulmonary hypertension. Prostaglandins Leukot Essent Fat Acids. (2001) 64:197–202. 10.1054/plef.2001.0261 11334556

[12] Sun D Yang W Wang Z Gao B. Efficacy of beraprost sodium combined with sildenafil and its effects on vascular endothelial function and inflammation in patients experiencing left heart failure complicated with pulmonary arterial hypertension. Med Sci Monit. (2021) 27:e928413. 10.12659/MSM.928413 33531453 PMC7869411

[13] Galiè N Humbert M Vachiéry JL Vizza CD Kneussl M Manes A. Effects of beraprost sodium, an oral prostacyclin analogue, in patients with pulmonary arterial hypertension: a randomized, double-blind, placebo-controlled trial. J Am Coll Cardiol. (2002) 39:1496–502. 10.1016/S0735-1097(02)01786-2 11985913

[14] Wang L Zhu X Zhao LP Wang M Liu X Chen Y. Effect of beraprost on pulmonary hypertension due to left ventricular systolic dysfunction. Medicine. (2019) 98:e14965. 10.1097/MD.0000000000014965 31008926 PMC6494404

[15] Takenaka M Iio A Sato R Sakamoto T Kurumatani H. A double-blind, placebo-controlled, multicenter, prospective, randomized study of beraprost sodium treatment for cats with Chronic Kidney Disease. J Vet Intern Med. (2018) 32:236–48. 10.1111/jvim.14839 29131397 PMC5787173

[16] Grubb T Sager J Gaynor JS Montgomery E Parker JA Shafford H Tearney C. 2020 AAHA anesthesia and monitoring guidelines for dogs and cats^*^. J Am Anim Hosp Assoc. (2020) 56:59–82. 10.5326/JAAHA-MS-7055 32078360

[17] Yuchi Y Suzuki R Kanno H Teshima T Matsumoto H Koyama H. Right ventricular myocardial adaptation assessed by two-dimensional speckle tracking echocardiography in canine models of Chronic Pulmonary Hypertension. Front Vet Sci. (2021) 8:727155. 10.3389/fvets.2021.727155 34485446 PMC8415444

[18] Hori Y Uchide T Saitoh R Thoei D Uchida M Yoshioka K. Diagnostic utility of NT-proBNP and ANP in a canine model of chronic embolic pulmonary hypertension. Vet J. (2012) 194:215–21. 10.1016/j.tvjl.2012.03.022 22578688

[19] Hosaka K Iwagaya Y Suzuki H Kojima T Kudo M Yoshinaka I. Acute and subacute toxicity study with beraprost sodium in dogs. Clin Rep. (1989) 23:3456–91.

[20] Ichino M Suzuki H Shibanushi T Kojima T Nakamura T Kudo M. Chronic toxicity study with beraprost sodium (PGI_2_) by the oral administration for 12 months in beagle dogs. Clin Rep. (1989) 23:3561–94.

[21] Takenaka M Takashima K Kurumatani H Ida N Sato R Yamane Y. Safety assessment of a prostacyclin derivative (Beraprost Sodium) in healthy cats. J Anim Clin Med. (2011) 20:131–9. 10.11252/dobutsurinshoigaku.20.131

[22] Acierno MJ Brown S Coleman AE Jepson RE Papich M Stepien RL. ACVIM consensus statement: guidelines for the identification, evaluation, and management of systemic hypertension in dogs and cats. J Vet Intern Med. (2018) 32:1803–22. 10.1111/jvim.15331 30353952 PMC6271319

[23] Visser LC Ciccozzi MM Sintov DJ Sharpe AN. Echocardiographic quantitation of left heart size and function in 122 healthy dogs: a prospective study proposing reference intervals and assessing repeatability. J Vet Intern Med. (2019) 33:1909–20. 10.1111/jvim.15562 31313382 PMC6766555

[24] Rishniw M Caivano D Dickson D Vatne L Harris J Matos JN. Two-dimensional echocardiographic left- atrial-to-aortic ratio in healthy adult dogs: a reexamination of reference intervals. J Vet Cardiol. (2019) 26:29–38. 10.1016/j.jvc.2019.11.001 31794915

[25] Cornell CC Kittleson MD Della Torre P Häggström J Lombard CW Pedersen HD. Allometric scaling of M-mode cardiac measurements in normal adult dogs. J Vet Intern Med. (2004) 18:311–21. 10.1111/j.1939-1676.2004.tb02551.x 15188817

[26] Schiller NB Shah PM Crawford M DeMaria A Devereux R Feigenbaum H. Recommendations for quantitation of the left ventricle by two-dimensional echocardiography. J Am Soc Echocardiogr. (1989) 2:358–67. 10.1016/S0894-7317(89)80014-8 2698218

[27] Gustafson DL Bailey DB. Cancer Chemotherapy, in Withrow MacEwen's Small Animal Clinical Oncology, eds. Vali D. M. Douglas T. H. Liptak J. M. (Elsevier), 182–208. 10.1016/B978-0-323-59496-7.00012-8

[28] Visser LC Scansen BA Schober KE Bonagura JD. Echocardiographic assessment of right ventricular systolic function in conscious healthy dogs: repeatability and reference intervals. J Vet Cardiol. (2015) 17:83–96. 10.1016/j.jvc.2014.10.003 25547662

[29] Rudski LG Lai WW Afilalo J Hua L Handschumacher MD Chandrasekaran K. Guidelines for the echocardiographic assessment of the right heart in adults: a report from the American Society of Echocardiography. Endorsed by the European Association of Echocardiography, a registered branch of the European Society of Cardiology, and the Canadian Society of Echocardiography. J Am Soc Echocardiogr. (2010) 23:685–713. 10.1016/j.echo.2010.05.010 20620859

[30] Gentile-Solomon JM Abbott JA. Conventional echocardiographic assessment of the canine right heart: reference intervals and repeatability. J Vet Cardiol. (2016) 18:234–47. 10.1016/j.jvc.2016.05.002 27453517

[31] Visser LC Im MK Johnson LR Stern JA. Diagnostic value of right pulmonary artery distensibility index in dogs with pulmonary hypertension: comparison with doppler echocardiographic estimates of pulmonary arterial pressure. J Vet Intern Med. (2016) 30:543–52. 10.1111/jvim.13911 26893108 PMC4913611

[32] Yuchi Y Suzuki R Teshima T Matsumoto H Koyama H. Utility of tricuspid annular plane systolic excursion normalized by right ventricular size indices in dogs with postcapillary pulmonary hypertension. J Vet Intern Med. (2021) 35:107–19. 10.1111/jvim.15984 33277735 PMC7848373

[33] Caivano D Dickson D Pariaut R Stillman M Rishniw M. Tricuspid annular plane systolic excursion-to-aortic ratio provides a bodyweight-independent measure of right ventricular systolic function in dogs. J Vet Cardiol. (2018) 20:79–91. 10.1016/j.jvc.2018.01.005 29503235

[34] Visser LC Sintov DJ Oldach MS. Evaluation of tricuspid annular plane systolic excursion measured by two-dimensional echocardiography in healthy dogs: repeatability, reference intervals, and comparison with M-mode assessment. J Vet Cardiol. (2018) 20:165–74. 10.1016/j.jvc.2018.04.002 29724583

[35] Suzuki R Yuchi Y Kanno H Saito T Teshima T Matsumoto H. Pulmonary vascular resistance estimated by echocardiography in dogs with myxomatous mitral valve disease and pulmonary hypertension probability. Front Vet Sci. (2021) 8:771726. 10.3389/fvets.2021.771726 34765671 PMC8576378

[36] Lewis JF Kuo LC Nelson JG Limacher MC Quinones MA. Pulsed Doppler echocardiographic determination of stroke volume and cardiac output: clinical validation of two new methods using the apical window. Circulation. (1984) 70:425–31. 10.1161/01.CIR.70.3.425 6744546

[37] Yuchi Y Suzuki R Teshima T Matsumoto H Koyama H. Right ventricular systolic and diastolic function assessed by two-dimensional speckle tracking echocardiography in dogs with myxomatous mitral valve disease. J Vet Med Sci. (2021) 83:1918–27. 10.1292/jvms.21-0195 34732606 PMC8762426

[38] Suzuki R Matsumoto H Teshima T Koyama H. Clinical assessment of systolic myocardial deformations in dogs with chronic mitral valve insufficiency using two-dimensional speckle-tracking echocardiography. J Vet Cardiol. (2013) 15:41–9. 10.1016/j.jvc.2012.09.001 23429036

[39] Suzuki R Matsumoto H Teshima T Koyama H. Effect of age on myocardial function assessed by two-dimensional speckle-tracking echocardiography in healthy beagle dogs. J Vet Cardiol. (2013) 15:243–52. 10.1016/j.jvc.2013.07.001 24054982

[40] Kanda Y. Investigation of the freely available easy-to-use software “EZR” for medical statistics. Bone Marrow Transplant. (2013) 48:452–8. 10.1038/bmt.2012.244 23208313 PMC3590441

[41] Feldhütter EK Domenech O Vezzosi T Tognetti R Sauter N Bauer A. Echocardiographic reference intervals for right ventricular indices, including 3-dimensional volume and 2-dimensional strain measurements in healthy dogs. J Vet Intern Med. (2021) 36:8–19. 10.1111/jvim.16331 34874066 PMC8783368

[42] Kellihan HB Stepien RL. Pulmonary hypertension in canine degenerative mitral valve disease. J Vet Cardiol. (2012) 14:149–64. 10.1016/j.jvc.2012.01.001 22364721

[43] Borgarelli M Abbott J Braz-Ruivo L Chiavegato D Crosara S Lamb K. Prevalence and prognostic importance of pulmonary hypertension in dogs with myxomatous mitral valve disease. J Vet Intern Med. (2015) 29:569–74. 10.1111/jvim.12564 25818210 PMC4895522

[44] Amundsen BH Helle-Valle T Edvardsen T Torp H Crosby J Lyseggen E. Noninvasive myocardial strain measurement by speckle tracking echocardiography: validation against sonomicrometry and tagged magnetic resonance imaging. J Am Coll Cardiol. (2006) 47:789–93. 10.1016/j.jacc.2005.10.040 16487846

